# Progesterone receptor integrates the effects of mutated MED12 and altered DNA methylation to stimulate RANKL expression and stem cell proliferation in uterine leiomyoma

**DOI:** 10.1038/s41388-018-0612-6

**Published:** 2018-12-11

**Authors:** Shimeng Liu, Ping Yin, Stacy A. Kujawa, John S. Coon, Ijeoma Okeigwe, Serdar E. Bulun

**Affiliations:** Division of Reproductive Science in Medicine, Department of Obstetrics and Gynecology, Feinberg School of Medicine, Northwestern University, Chicago, IL 60611 USA

**Keywords:** Mutation, Hormone receptors, DNA methylation, Cytokines, Histone post-translational modifications

## Abstract

Progesterone and its receptor, PR, are essential for uterine leiomyoma (LM, a.k.a., fibroid) tumorigenesis, but the underlying cellular and molecular mechanisms remain unclear. The receptor activator of NF-κB (RANKL) was recently identified as a novel progesterone/PR-responsive gene that plays an important role in promoting LM growth. Here, we used RANKL as a representative gene to investigate how steroid hormone, genetic, and epigenetic signals are integrated to regulate LM stem cell (LSC) function. We demonstrated that RANKL specifically upregulates LSC proliferation through activation of Cyclin D1. RANKL gene transcription was robustly induced by the progesterone agonist R5020, leading to a dramatically higher RANKL expression in LM compared to adjacent myometrial (MM) tissue. MethylCap-Seq revealed a differentially methylated region (DMR) adjacent to the distal PR-binding site (PRBS) 87 kb upstream of the RANKL transcription start site. Hypermethylation of the DMR inhibited recruitment of PR to the adjacent PRBS. Luciferase assays indicated that the DMR and distal PRBS constitute a novel RANKL distal regulatory element that actively regulates RANKL expression. Furthermore, MED12 physically interacts with PR in LM tissue. The interaction between MED12 and PR, binding of PR and MED12 to PRBS, and RANKL gene expression are significantly higher in LM containing a distinct MED12 mutation (G44D) than in LM with wild-type MED12. In summary, our findings suggest that DNA methylation and MED12 mutation together constitute a complex regulatory network that affects progesterone/PR-mediated RANKL gene expression, with an important role in activating stem cell proliferation and fibroid tumor development.

## Introduction

Uterine leiomyomas (LM, a.k.a., fibroids) represent the most common gynecological tumors in women. By age 50, about 80% of women will develop at least one LM, and 15–30% will develop severe symptoms, including excessive uterine bleeding, recurrent pregnancy loss, and pelvic pain; these symptoms may mimic or mask malignant tumors [1]. Approximately 200,000 hysterectomies and 30,000 myomectomies are performed to treat LM in the US annually, costing up to $34.4 billion [2]. Considering the heavy socioeconomic burden and associated long-term health problems, there is an urgent need to develop non-surgical treatments for LM.

Progesterone (P4) and its receptor, PR, play essential roles in LM development; however, the cellular and molecular mechanisms responsible remain unclear [3]. Anti-progestins have proven useful in the medical management of LM; however, long-term usage is limited by their side effects and rapid tumor recurrence after treatment cessation [3, 4]. Thus, understanding the molecular mechanisms underlying P4/PR action in LM pathogenesis is critical for the development of targeted and effective therapies for this disease.

Receptor activator of nuclear factor κB ligand (RANKL), a P4/PR target gene, recently became a key actor in oncology [5]. The molecular axis of RANKL and its receptor RANK is involved in all stages of tumorigenesis [5]. In mouse mammary gland, progestins substantially stimulate RANKL expression in PR-positive cells, leading to paracrine activation of PR-negative mammary stem cells and progression of progestin-driven mammary cancer [6–8]. Later studies in humans have demonstrated the positive correlation between serum P4 levels and RANKL mRNA and protein levels in normal and malignant breast tissues [9, 10]. We recently demonstrated that blocking the RANKL/RANK pathway inhibits steroid hormone-mediated LM development in a xenograft mouse model, indicating that this pathway plays a critical role in LM pathogenesis [11]. However, the mechanisms responsible for the hormone responsiveness of RANKL gene expression and the role of RANKL in LM formation require further elucidation.

Epigenetic alterations, such as DNA methylation, play crucial roles in regulating gene expression and disease progression [12]. DNA methylation interferes with the interactions between DNA and specific transcription factors (TFs) and chromatin proteins [13]. Abnormal DNA methylation and expression of its regulatory enzymes have been reported in LM [14, 15]. However, the role of DNA methylation in P4/PR-mediated transcriptional regulation in LM has not been explored.

Mutation of mediator complex subunit 12 (MED12) is the most prevalent mutation (mut-MED12) in LM, occurring in over 70% of all LMs [16]. LM-associated mut-MED12 may alter its interactions with proteins involved in transcriptional co-activator pathways [17]. For instance, mut-MED12 disrupts the MED12-Cyclin C binding interface, leading to a loss of mediator-associated CDK activity [18]. Introduction of the most frequent mut-MED12 subtype, G44D, in mice causes LM development in the presence of estradiol (E2) and P4. So far, the hormone-dependent mechanism whereby mut-MED12 causes LM formation remains unclear.

Here, we used RANKL as a representative P4/PR target gene to study the genetic and epigenetic mechanisms underlying dysregulated RANKL expression in LM, which will also shed mechanistic light on our understanding of RANKL gene regulation and PR action in breast cancer and other steroid-responsive tumors [5].

## Results

### RANKL is highly overexpressed in LM versus adjacent normal MM

**Fig. 1:**
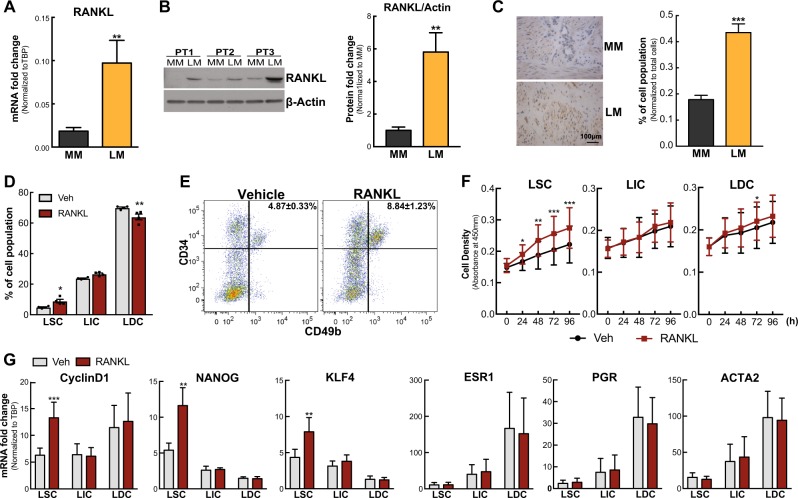
RANKL stimulates LSC proliferation. **a** Histogram showing mRNA levels of RANKL in LM and MM tissues (means ± SEM, *n* = 43, **P < 0.01, paired *t*-test). **b** Left panel, representative immunoblot showing RANKL protein levels in LM and MM tissues; right panel, ImageJ quantification of immunoblots (means ± SEM, *n* = 6, **P < 0.01, paired *t*-test). PT: patient. **c** LM and MM tissue sections were stained with anti-RANKL antibody. Left panel, representative images of immunohistochemical staining of RANKL in MM and LM tissues; right panel, percentage of RANKL-positive cells among total cells counted (means ± SEM, *n* = 10, ***P < 0.001, paired *t*-test). **d** Percentage of LM cell populations isolated from tissue explants after 24 h incubation with vehicle (0.1% BSA in PBS) or RANKL (100 ng/ml) (means ± SEM, *n* = 4, **P < 0.01 and *P < 0.05, paired *t*-test). LM cells were stained with anti-CD45 (depleted hematopoietic cells), anti-CD34, anti-CD49b, and PI (depleted dead cells) and analyzed by the LSR Fortessa system. **e** Representative FACS scattergram for panel **d**. **f** Line graph showing the proliferation rate of each LM cell population treated with vehicle (0.1% BSA in PBS) or RANKL (100 ng/ml) for different time indicated on the graph (means ± SEM, *n* = 3, ***P < 0.001, **P < 0.01, and *P < 0.05, paired *t*-test). **g** Regulation of proliferation, stem cell, and differentiation related gene expression by RANKL treatment in each LM cell population determined by real-time PCR (means ± SEM, *n* = 5, **P < 0.01 and ***P < 0.001, paired *t*-test)

We examined in vivo RANKL mRNA levels in 86 matched MM and LM tissues (*n* = 43 patients). RANKL mRNA was significantly higher in LM compared with matched MM tissues (Fig. 1a). Immunoblot analysis showed that the RANKL protein level was also markedly upregulated in LM (Fig. 1b). Immunohistochemistry revealed more intense and abundant levels of RANKL protein distributed in the cytoplasm and intercellular matrix in LM tissues (Fig. 1c).

### RANKL stimulates LM stem cell proliferation

There are three distinct cell populations in LM based on CD34 and CD49b expression: CD34^+^/CD49b^+^ (LSC, LM stem/progenitor cells), CD34^+^/CD49b^−^ (LIC, intermediate cells), and CD34^−^/CD49b^−^ (LDC, differentiated cells) [19]. Based on RANKL’s established function in breast cancer, we hypothesized that RANKL plays a critical role in LM pathogenesis by stimulating LSC proliferation. Hence, we treated LM tissue explants with synthetic human RANKL or vehicle for 24 h and analyzed the percentage of LM cell populations. RANKL treatment markedly expanded the LSC population compared to control (8.84 ± 1.23% vs 4.87 ± 0.33%) but decreased the percentage of LDC population (64.97 ± 1.88% vs 69.96 ± 0.53%), whereas the LIC population was not significantly affected (Fig. 1d, e).

To evaluate the mechanism underlying the percentage change of LM cell populations after RANKL treatment, we conducted proliferation (CCK8 assay) and apoptosis (Annexin V staining) assays. RANKL specifically increased LSC proliferation (Fig. 1f) without affecting cell survival (Supplementary Figure S1). Furthermore, RANKL significantly increased expression of proliferation gene (Cyclin D1) and stem cell factors (KLF4 and NANOG) particularly in LSC, but did not affect the expression levels of differentiation markers (ESR1, PGR, and ACTA2) (Fig. 1g). These findings indicate that RANKL promotes LM growth through inducing proliferation selectively in the stem cell population.

### RANKL expression is differentially regulated by P4/PR signaling in LM versus MM

**Fig. 2:**
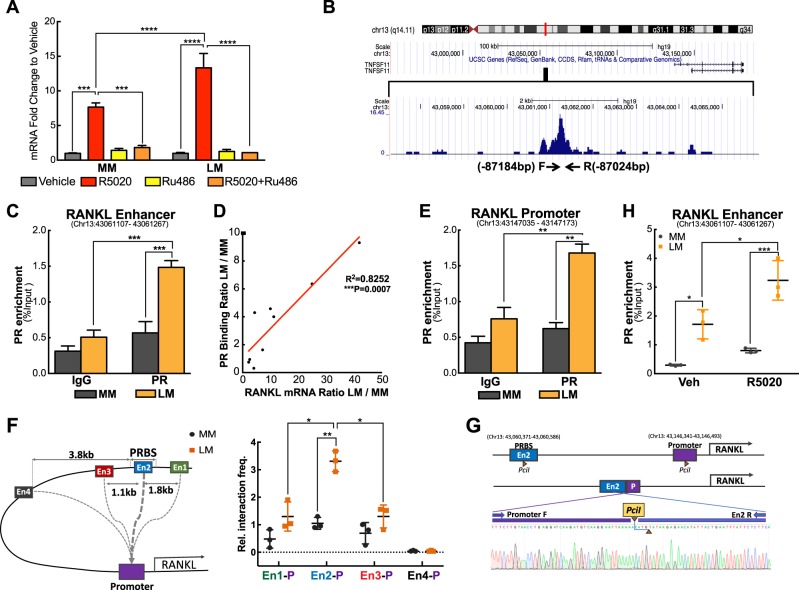
Differential RANKL regulation by P4/PR signaling in MM and LM. **a** Relative RANKL mRNA levels normalized to vehicle-treated cells were determined by real-time PCR in MM and LM tissue explants treated with R5020 (10^−7^ M) or vehicle (ethanol) in the presence or absence of RU486 (10^−^^6^M) for 48 h (means ± SEM, *n* = 6, ***P < 0.001 and ****P < 0.0001, paired two-way ANOVA). **b** UCSC Genome Browser ChIP-Seq Track showing PR-binding peak located 87 kb upstream of the RANKL transcription start site in LM. PROMO (V3.0.2) revealed enriched SP-1 motif and half-PRE sites in the region. **c** ChIP-qPCR showing PR enrichment at the RANKL distal PR-binding site (PRBS, −87,184/−87,024 bp, Chr13: 43,061,107–43,061,267) (means ± SEM, *n* = 10, ***P < 0.001, paired two-way ANOVA). **d** Pearson correlation showing positive correlation between PR enrichment at the distal PRBS and RANKL mRNA levels. Data represents PR enrichment and RANKL mRNA levels in LM normalized to those in matched MM from nine subjects (*R*^2^ = 0.8252, ***P < 0.001, Pearson correlation). **e** ChIP-qPCR showing PR enrichment at the RANKL proximal promoter (−1256/−1118 bp, Chr13: 43,147,035–43,147,173) (mean ± SEM, *n* = 10, **P < 0.01, paired two-way ANOVA). **f** Left panel: illustration of examined chromatin interactions of the RANKL promoter and distal PRBS. The promoter region was designed as the “bait” and four “interrogated fragments” were showed around the PRBS with En2 overlapped with PRBS. All investigated regions were located within 150 bp of *PciI* sites. Right panel: 3C-qPCR (dot plot) showing RANKL promoter and distal enhancer interactions in MM and LM primary cells (means ± SEM, *n* = 3, *P < 0.05, **P < 0.01, paired two-way ANOVA). Results are presented as relative interaction frequencies normalized by GAPDH as an internal control. P: promoter; En1, En2, En3, and En4 represent four DNA fragments evaluated. **g** Schematic diagram of RANKL promoter and En2 regions following *PciI* excision and ligation. Sequence chromatogram of excised band with regions of interest denoted. **h** PR ChIP-qPCR showing PR recruitment to the distal PRBS in primary MM and LM cells exposed to vehicle or R5020 for 1 h (means ± SEM, *n* = 3, *P < 0.05 and ***P < 0.001, paired two-way ANOVA)

Our finding that RANKL plays a critical role in regulating LSC function and LM tumorigenesis prompted us to further explore the mechanism underlying dysregulated RANKL gene expression in LM [11]. RANKL is a known P4/PR target gene in mammary glands [20, 21]; therefore, we postulated that the RANKL gene responds to P4/PR signaling differentially between LM and MM tissues, leading to higher RANKL expressions in LM. To test this, we quantified RANKL mRNA levels in MM and LM tissue explants treated with the P4 agonist R5020 for 48 h. R5020 increased RANKL expression in both LM and MM explants, but the fold change was significantly higher in LM, suggesting RANKL gene transcription activity is more sensitive to R5020 in LM cells than in MM cells (Fig. 2a, 13.10 ± 1.02 LM vs 7.75 ± 0.63 MM). The R5020-mediated induction of RANKL expression in both tissues was completely blocked by co-treatment with RU486 (P4 antagonist) in explants (Fig. 2a) or PR knockdowns (Supplementary Figure S2A) in cells, suggesting that R5020 regulates RANKL expression through PR.

We examined our previously published PR ChIP-sequencing (ChIP-Seq) data in LM cells and found multiple PR-binding sites (PRBS) around RANKL gene locus [22]. A region 87 kb upstream (−87,360/−86,731 bp) of RANKL transcription start site (TSS) has been identified to display the strongest PR-binding activity among all binding sites (Fig. 2b) and is enriched for DNA-binding motifs of SP-1 and progesterone response element (PRE) half sites (PROMO V3.0.2), which are important for PR–chromatin interaction [23]. We designed four sets of primers covering the entire distal PRBS and performed ChIP-qPCR to examine the enrichment of PR binding using chromatin isolated from fresh-frozen LM and MM tissues. We detected higher PR-binding activity in LM than in MM on four sets of primers spanning Chr13: 43,060,931–43,061,560 (Fig. 2c and Supplementary Figure S2B). Regression analysis demonstrated that the enhanced PR-binding activity in this region was positively correlated with RANKL mRNA level (*R*^2^ = 0.8252), suggesting that the distal PRBS is an enhancer region that regulates RANKL transcription (Fig. 2d).

Using PR ChIP-Seq, we also detected PR-binding activity in the RANKL promoter region (−2200/−1000 bp). This region has strong gene transcription regulatory activity and is enriched with DNA-binding motifs of AP-1 and PRE half sites, which are important for PR recruitment to DNA [24–26]. To elucidate whether the −87kb distal PRBS was associated with the proximal promoter, we first determined whether PR was recruited to the promoter region in LM using ChIP-qPCR. We found that PR enrichment in the RANKL promoter (−1256/−1118 bp) was not only higher in LM versus MM tissue, but also positively correlated (*R*^2^ = 0.7407) with PR binding at the distal PRBS (Fig. 2e and Supplementary Figure S2C). Next, we performed chromatin conformation capture (3 C)-qPCR assay to further confirm the chromatin interaction between the distal PRBS and proximal promoter region. As shown in Fig. 2f, we evaluated four fragments (En1, En2, En3, and En4) around PRBS, and found the interaction strength was significantly stronger in LM versus MM with the highest interaction observed between promoter and En2, which is the region overlapped with the distal PRBS. No interaction was detected between the RANKL promoter and En4 region, which is 3.8 kb away from the distal PRBS. Sanger sequencing of the amplicon of promoter and En2 confirmed the expected ligation product from loop formation (Fig. 2g). This suggests that the distal PRBS regulates RANKL gene expression through interaction with the proximal promoter. PR was not recruited to the RPL30 housekeeping gene promoter, and IgG did not contain enriched chromatin in the RANKL or the RPL30 gene, indicating that PR enrichment in the RANKL regulatory region is specific (Supplementary Figure S2D).

To examine whether the enhanced PR binding around the RANKL gene in LM was due to differential PR expression levels between the two tissues, we measured PR mRNA and protein levels in the tissues used in the ChIP-qPCR. The PR mRNA level in LM was only marginally higher (1.3-fold) than in MM tissue (Supplementary Figure S2E), with the comparable PR protein level between MM and LM tissue chromatin (Supplementary Figure S2F). These findings suggest that the specific epigenetic context of the distal enhancer region, not the PR expression level, leads to increased recruitment of PR to the RANKL gene in LM tissue.

To determine whether PR-binding activity at the RANKL distal PRBS is ligand-dependent, primary LM and MM cells were treated with R5020 for 1 h followed by ChIP-qPCR. Consistent with our findings in LM and MM tissues, basal PR-binding affinity at the enhancer region was higher in LM versus MM cells (Fig. 2h). R5020 increased PR binding to the distal enhancer in both LM and MM cells; however, R5020-mediated PR enrichment was still higher in LM cells.

### Active histone marks are highly enriched in distal enhancer and proximal promoter regions of the RANKL gene

**Fig. 3:**
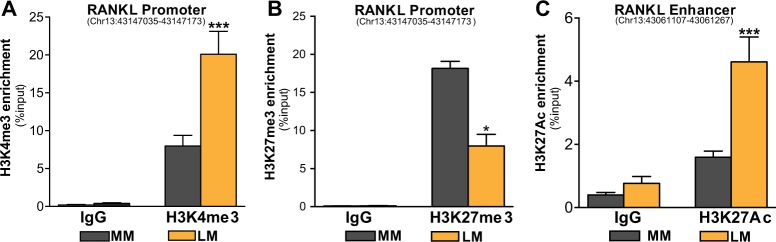
Differential histone modification patterns at the RANKL proximal promoter and distal PRBS in MM and LM tissues. ChIP-qPCR assay showing histone marks H3K4me3 (**a**) and H3K27me3 (**b**) enrichment at the RANKL proximal promoter, and H3K27Ac (**c**) enrichment at distal PRBS in MM and LM tissues (*n* = 5, means ± SEM, *P < 0.05 and ***P < 0.001, paired two-way ANOVA)

Post-translational modification of histone tails plays an important role in regulating gene transcription. High levels of H3K4me3 are commonly found in the promoters of actively transcribed genes, whereas H3K27me3 enrichment is associated with inactive promoters [27]. ChIP-qPCR of histone modifications revealed that H3K4me3 enrichment at the RANKL promoter was higher in LM than in MM, whereas H3K27me3 levels were higher in MM (Fig. 3a, b). In the distal PRBS, H3K27Ac (active enhancer mark) was more highly enriched in LM versus MM (Fig. 3c), confirming the enhancer characteristic of this region. These histone modification patterns not only support our observation of higher RANKL transcription in LM, but also indicate a more accessible chromatin structure adjacent to the RANKL gene in LM.

### DNA methylation mediates RANKL gene expression

**Fig. 4:**
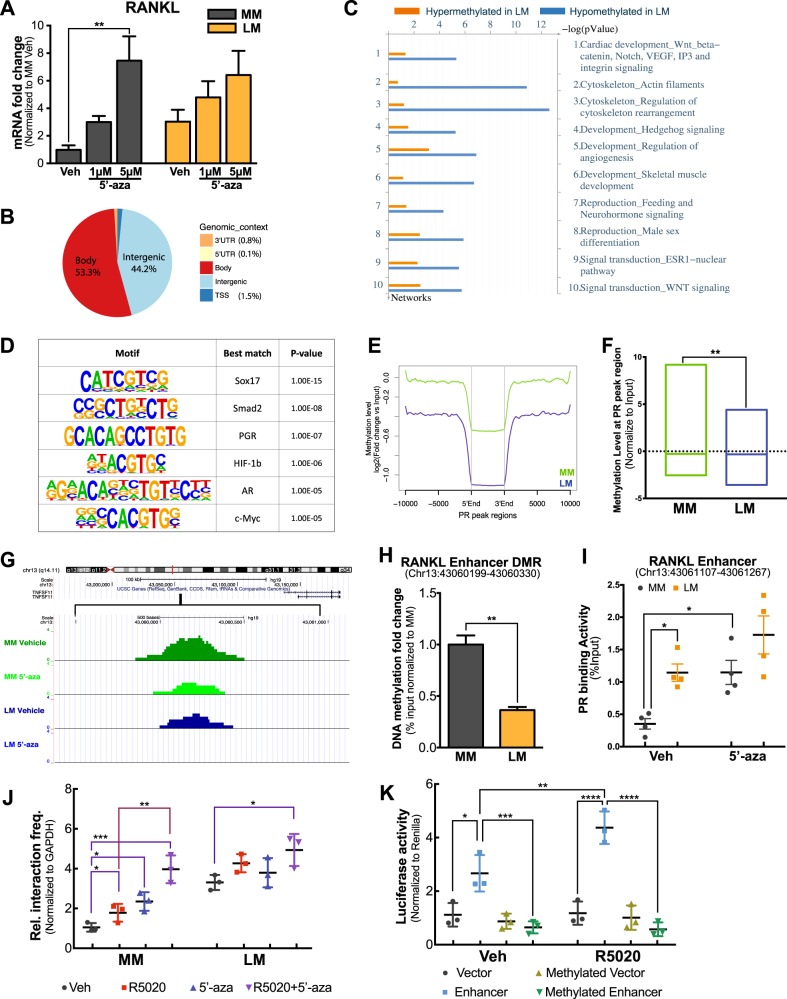
Effects of DNA methylation on RANKL transcription and PR association with the distal PRBS of the RANKL gene. **a** Histogram showing RANKL mRNA levels in primary MM and LM cells treated with or without 5′-aza for 4 days (means ± SEM, *n* = 5, **P < 0.01, paired one-way ANOVA). **b** Pie chart showing the genomic context of 21,086 differentially methylated regions (DMRs) identified using MethylCap-Seq. **c** Network enrichment analysis (Metacore) of DMRs. **d** Motif analysis (Homer) of all DMRs between LM and MM. **e** Genome-wide PR-binding regions in LM are more hypomethylated in LM versus MM. All PR-binding sites identified by PR ChIP-Seq in LM tissue were scanned from 10 kb upstream to 10 kb downstream of the binding region and the average DNA methylation levels at these regions were determined using the MethylCap-Seq data of untreated MM (green) and LM (blue) cells. **f** DNA regions with higher PR-binding activity had lower DNA methylation levels in LM compared with MM. Box plot showing normalized DNA methylation levels (log2(RPKM_IP_ + 1) – log2(RPKM_Input_ + 1)) at 2662 PR-binding regions with higher PR enrichment in LM versus MM tissue (**P < 0.01, paired *t*-test). Line within the box indicates median DNA methylation levels of all regions. **g** DNA methylation levels of MM and LM cells exposed to 5ʹ-aza (5 µM) or DMSO were evaluated using MethylCap-Seq. Representative UCSC Genome Browser Track view showing a DMR adjacent to the distal PRBS. **h** MethylCap-qPCR confirmed the higher DNA methylation level of DMR (−88,092 bp/−87,961 bp, Chr13: 43,060,199–43,060,330) adjacent to the distal PRBS of RANKL gene in MM versus LM tissue. Bar plots showing relative DNA methylation levels in LM after normalization to MM using genomic DNA from five different subjects (means ± SEM, *n* = 5, **P < 0.01, paired *t*-test). **i** Dot plot of ChIP-qPCR of PR association with the RANKL gene distal PRBS in primary MM and LM cells exposed to 5ʹ-aza (5 μM, 96 h) or vehicle (DMSO) (means ± SEM, *P < 0.05, paired two-way ANOVA, *n* = 3). All groups were stimulated by R5020 (10^−^^7 ^M, 1 h) prior to harvesting. **j** Dot plot showing 3C-qPCR results of the RANKL promoter and distal PRBS (En2 fragment shown in Fig. 2f) interaction in MM and LM primary cells treated with 5ʹ-aza (5 μM, 96 h) or vehicle (DMSO) in the presence or absence of R5020 (10^−^^7^ M, 1 h) (means ± SEM, *n* = 3, *P < 0.05, **P < 0.01, ***P < 0.001, paired two-way ANOVA). **k** DNA fragment spanning distal PRBS and the DMR (−88,347 bp/−86,851 bp, chr13: 43,059,944–43,061,440, 1496 bp, designated as RDRE) was cloned into a CpG-free luciferase construct. RDRE-containing or empty vector (control) were treated with or without methylation reagents in vitro. LM primary cells were transiently transfected with the treated plasmids, exposed to vehicle (ethanol) or 10^−7 ^M R5020 for 48 h, and harvested for luciferase assay (*n* = 3, means ± SEM, *P < 0.05, **P < 0.01, ***P < 0.001, ****P < 0.0001, paired two-way ANOVA)

Studies have shown that DNA methylation at the CpG island adjacent to the RANKL promoter mediates RANKL expression in bone and mammary gland tissues [28, 29]. To evaluate whether RANKL transcription is regulated by DNA methylation in MM and LM, we treated primary LM and MM cells with the DNA methyltransferase inhibitor 5-aza-2′-deoxycytidine (5′-aza) for 4 days. We found that RANKL mRNA levels in MM cells were more dramatically increased by 5′-aza compared with LM (Fig. 4a). The mRNA levels of RANK and osteoprotegerin (OPG, a decoy RANKL receptor) were not affected by 5′-aza, suggesting that DNA methylation specifically regulates RANKL expression in MM and LM cells (Supplementary Figures S3A and B). In addition, we demonstrated that 5′-aza consistently induced RANKL expression in MM cells in the presence of the protein synthesis inhibitor cycloheximide, suggesting that 5′-aza regulates RANKL expression by directly modulating its DNA methylation (Supplementary Figure S3C). To determine whether 5′-aza-mediated RANKL expression through altering the CpG island DNA methylation as previously reported [28, 29], we performed HumanMethylation450K array. No DNA methylation change was detected for all probes around RANKL gene locus (Supplementary Figure S3E).

### DNA methylation potentially alters global progesterone responsiveness in LM

To define the mechanism underlying 5ʹ-aza-mediated RANKL expression change, we isolated genomic DNA from LM and MM tissues and performed MethylCap-Seq to profile genome-wide methylation landscapes of MM and LM cells treated with or without 5ʹ-aza. We discovered 21,086 differentially methylated regions (DMRs) between untreated MM and LM cells. Intriguingly, 44.23% of DMRs were found to be located in intergenic regions (Fig. 4b). Network enrichment analysis indicated that DMR-associated genes, especially those close to hypomethylated regions in LM, were highly enriched for genes encoding signaling components that control the Wnt/β-catenin pathway, cytoskeleton regulation, skeletal muscle development, and the estrogen receptor 1 (ESR1) nuclear pathway (Fig. 4c), which have been shown to play crucial roles in LM development [30–32].

Motif enrichment analysis of all DMRs between LM and MM showed that PREs (PR and androgen receptor (AR) bind to essentially identical motifs), together with several other critical TF binding elements such as HIF-1b and Smad2, were highly enriched at DMRs (Fig. 4d). Since PREs were enriched around DMRs, we investigated the methylation status of genome-wide PRBSs by integrating MethylCap-Seq data with PR ChIP-Seq data in matched MM and LM tissues. We found that DNA methylation levels at PRBSs in LM were lower versus those in MM (Fig. 4e and Supplementary Table 1), particularly in regions with higher PR-binding activity in LM (Fig. 4f and Supplementary Table 2).

### The RANKL gene distal enhancer region is hypomethylated and controls RANKL transcription by interfering with PR action

Our findings suggested that DNA methylation may contribute to the global difference in progesterone responsiveness of LM and MM through interference of PR-binding activity. We specifically examined DMRs adjacent to the RANKL gene locus and discovered a hypermethylated region located 500 bp upstream of the distal PRBS in MM versus LM cells, which was de-methylated after 5ʹ-aza treatment, but no methylation difference was detected adjacent to the RANKL promoter (Fig. 4g and Supplementary Figure S3D). Using MethylCap-qPCR, we validated our findings using genomic DNA isolated directly from matched LM and MM tissues of five subjects. DNA methylation levels at this DMR was significantly lower in LM versus MM tissues (Fig. 4h).

To test whether DNA methylation affects PR-binding activity at the distal PRBS of the RANKL gene, we treated primary MM and LM cells with 5ʹ-aza (5 µM) or vehicle (DMSO) for 4 days and examined PR recruitment to this region. In vehicle-treated cells, PR-binding activity was significantly higher in LM than in MM cells (Fig. 4i). Consistent with the effect of demethylation on RANKL transcription, 5ʹ-aza robustly enhanced PR binding in MM but not in LM cells (Fig. 4i), resulting in similar binding activity between LM and MM under 5ʹ-aza treatment condition.

Furthermore, we evaluated the effects of 5ʹ-aza and R5020 treatment on chromatin interaction activity between the distal PRBS and the RANKL promoter using 3C-qPCR. In MM, the looping activity between these two regions in vehicle-treated cells was weak, increased slightly with either 5ʹ-aza or R5020 treatment, and peaked when the two treatments were combined (Fig. 4j). Consistent with the results shown in Fig. 2f, in vehicle-treated cells, the interaction activity was higher in LM versus MM cells and 5ʹ-aza alone did not significantly change the interaction strength in LM (Fig. 4j). Consequently, 5ʹ-aza triggered the interaction strength between the distal PRBS and RANKL promoter in MM to the levels observed in LM, which is consistent with 5ʹ-aza-mediated RANKL mRNA expression (Fig. 4a) and PR recruitment towards the distal PRBS (Fig. 4i).

We recently reported that RANKL is preferentially expressed and specifically upregulated by R5020 in LIC [11], we went on investigating whether the same mechanism (DNA methylation difference in the DMR adjacent to the distal PRBS of RANKL gene) was also involved. We examined the DNA methylation levels and the effects of 5ʹ-aza treatment on RANKL expression in each LM cell population. DNA methylation level in the DMR was significantly lower in LIC versus LDC, accompanied with higher RANKL mRNA levels in LIC (Supplementary Figure S3F). 5ʹ-Aza stimulated RANKL expression more robustly in LDC (Supplementary Figure S3G). We did not detect significant correlation between DNA methylation and RANKL expression in LSC, which is deficient of PR [19].

Together, our findings indicate that the novel DMR upstream of the distal PRBS may mediate RANKL expression. We designated the DNA fragment spanning the novel DMR and the distal PRBS as the RANKL distal regulatory element (RDRE). To determine whether the RDRE has a regulatory function in RANKL gene transcription, we cloned the 1496 bp RDRE (chr13: 43,059,944–43,061,440) into a CpG-free luciferase construct and performed luciferase reporter assays [33]. As shown in Fig. 4k, the vector carrying the RDRE showed significant promoter-enhancing activity in vehicle-treated cells, which was further increased by R5020 treatment. In vitro methylation of this fragment completely blocked its promoter-enhancing activity. These results indicate that RDRE has progestin-inducible transcriptional regulatory activity and can be blocked by DNA methylation.

### MED12 mutation status influences RANKL transcription

Mutations in MED12 alter its interaction with transcriptional co-activators and induce E2 + P4-dependent LM formation [17, 34]; this prompted us to hypothesize that MED12 mutations dysregulate its interaction with PR, leading to increased RANKL transcription in LM. Due to RANKL’s effect on LSC function, we investigated whether mut-MED12 affects RANKL expression and PR recruitment to the distal PRBS. To minimize the potential variation caused by different mutations of MED12, we focused on the most common mutation type, i.e., G44D mut-MED12 [16].

**Fig. 5:**
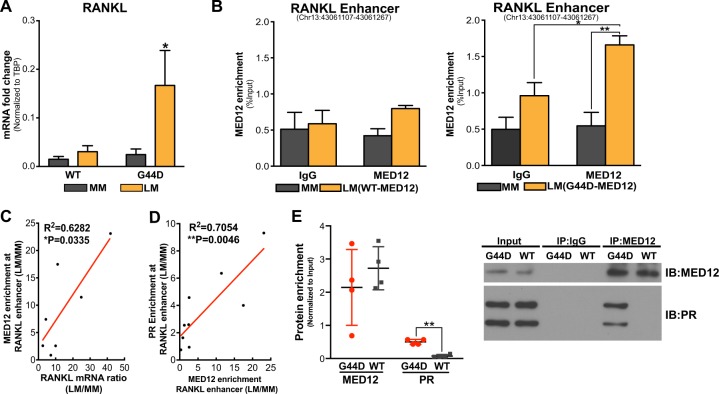
**MED12 mutation status influences RANKL transcription.** **a** RANKL mRNA levels in wild type (WT) (*n* = 12) or G44D MED12 mutation-containing (*n* = 12) LM and their matched MM tissues (mean ± SEM, *P < 0.05, paired two-way ANOVA). RANKL mRNA levels were normalized by TBP expression. **b** ChIP-qPCR of MED12 recruitment towards the distal PRBS of RANKL gene using chromatin isolated from fresh-frozen MM and LM tissues. Results were stratified based on MED12 mutation status, including four subjects expressing WT-MED12 and nine subjects bearing G44D MED12 mutation (means ± SEM, *P < 0.05, **P < 0.01, paired two-way ANOVA). **c**, **d** Pearson correlation showing that MED12 enrichment at RANKL distal PRBS positively correlates with RANKL expression level (**c**, *n* = 7, *R*^2^ = 0.6282, *P < 0.05, Pearson correlation) and PR-binding activity in LMs carrying the G44D MED12 mutation (**d**, *n* = 9, *R*^2^ = 0.7054, **P < 0.01, Pearson correlation). Data represent MED12 enrichment and RANKL mRNA levels in LM normalized to those in matched MM. **e** IP-IB assay showing higher physical interaction activity between MED12 and PR in LMs expressing G44D MED12 mutation compared with those expressing WT-MED12. Whole-cell protein extracts were immunoprecipitated with either MED12 or IgG followed by immunoblotting for PR and MED12. Left panel: ImageJ quantification of immunoblots (means ± SEM, *n* = 3, **P < 0.01, paired *t*-test); Right panel: representative blot

We observed that RANKL mRNA levels and recruitment of MED12 in the RANKL distal PRBS were significantly higher in LM tissues carrying G44D mut-MED12 than those expressing wild-type (WT) MED12 (Fig. 5a, b) (MM tissues always express WT-MED12). Regression analysis demonstrated that MED12-binding activity at the enhancer region positively correlated with RANKL expression (Fig. 5c, R^2^ = 0.6282) and PR-binding affinity in this region (Fig. 5d, *R*^2^ = 0.7054) in LM with G44D mut-MED12. Supporting the pro-proliferation role of RANKL in LSC, we detected a marginally larger (*P* = 0.0551) LSC population in LM harboring G44D mutation versus wild type (WT) LM (Supplementary Figures S4). In addition, immunoprecipitation-immunoblot (IP-IB) analysis showed that PR interacted with MED12 and the interaction was stronger in LM-bearing G44D mut-MED12 (Fig. 5e). These findings suggest that MED12 mutation status influences RANKL transcription and its interaction with PR.

## Discussion

**Fig. 6:**
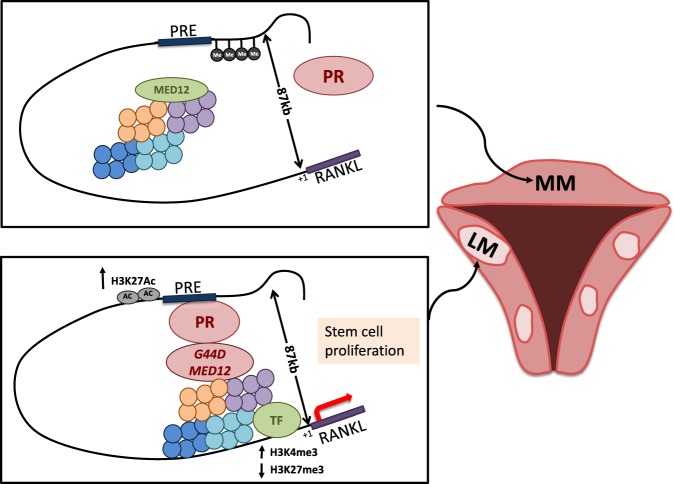
Schematic of the proposed RANKL gene regulatory model in MM and LM. In MM, higher DNA methylation at RDRE blocks PR binding, resulting in low RANKL expression levels. In LM, RDRE is hypomethylated, leading to higher PR-binding activity. G44D-mutated MED12 further stabilizes PR binding at the RDRE

We uncovered a novel cis-regulatory element (RDRE) downstream of P4/PR signaling that regulates RANKL transcription. We demonstrated that RANKL expression is regulated by converging signals from steroid hormone (P4/PR), genetic factors (MED12 mutation), and epigenetic modifications (DNA methylation and histone modification) and contributes to LSC proliferation and tumor formation. We summarize our proposed model of P4/PR-mediated RANKL gene regulation in Fig. 6.

The RANKL/RANK pathway is known for its crucial role in paracrine signaling between stem and differentiated cells in breast cancer, where RANKL, released from PR-positive cells upon P4 stimulation, binds to its receptor on PR-negative progenitor cells and stimulates tumorigenesis through expanding the progenitor cell population [35]. RANKL is mainly expressed in LIC, whereas RANK is predominantly present in LSC, suggesting a similar paracrine action of RANKL/RANK in LM tumorigenesis [11]. Here, we found that RANKL treatment induces Cyclin D1 expression and cell proliferation specifically in LSC, leading to LSC expansion (Fig. 1d–g). The tumorigenic function of the RANKL/RANK signaling pathway was further supported by our findings that blocking this pathway inhibits in vivo steroid hormone (E2 and P4)-dependent LM growth in a xenograft mouse model [11].

Previous studies have demonstrated that RANKL transcription is directly regulated by DNA methylation at its promoter in bone and mammary gland tissues [29, 36]. However, the same region was not differentially methylated between LM and MM, suggesting tissue specificity of methylation-mediated RANKL expression [37]. Using genome-wide MethylCap-Seq, we discovered a DMR adjacent to the distal PRBS identified previously via PR ChIP-Seq in LM cells [22]. In vitro demethylation of this region increased R5020-dependent recruitment of PR to the adjacent PRBS, whereas methylation repressed fused reporter gene luciferase activity, suggesting that the DMR functionally regulates PR access to its responsive element as well as its transcriptional activity. Methyl-CpG has been proposed to interfere with TF binding directly and indirectly [38]. The direct model posits that the specific TF sees a methyl-CpG as a mutation in its binding site and thus is unable to bind. The indirect model proposes that methylated DNA is bound by a nuclear protein(s), such as MeCP-1, which interrupts the particular TF’s interaction with its target gene. Our finding that the DMR resides outside but adjacent to the PRBS is compatible with the indirect model of transcriptional interference, although the detailed mechanism needs further investigation [38].

Furthermore, gene transcription is also controlled by extensive crosstalk and interactions between DNA methylation and histone modifications. For example, in embryonic stem cells, H3K4 methylation (including mono-, di-, and tri-methylation) can prevent the binding of DNA methylation machinery DNMT3L, resulting in lower methylation levels at nearby CpG sites [39]. Consistent with this notion, higher levels of active histone marks (H3K27Ac and H3K4me3) and lower levels of a repressive mark (H3K27me3) were detected in the RANKL distal enhancer region with lower DNA methylation in LM versus MM.

The human mediator complex was first identified through an intracellular ligand-dependent association with thyroid hormone receptor [40]. Since then, many nuclear receptors have been shown to interact with the mediator subunits [41]. For example, ESR1’s interaction with MED1 is required for ESR1-mediated gene transcription, E2/ESR1-dependent mammary progenitor/stem cell function, and breast cancer cell growth [42, 43]. To our knowledge, our study is the first to report the interaction of MED12 with PR. Importantly, we found that MED12-binding activity at the RANKL distal PRBS not only positively correlated with PR-binding affinity in this region but also positively correlated with RANKL expression. Furthermore, we demonstrated that mut-MED12 increases its interaction with PR, leading to higher RANKL expression in LM carrying mut-MED12. These observations suggest that PR-MED12 interaction in LM is functionally active in regulating P4/PR-mediated gene expression and mut-MED12 may affect chromatin interaction with PR, leading to preferential transcription of genes and pathways that favor fibroid tumorigenesis.

In support of our findings, previous researches have demonstrated that LM-associated MED12 mutations could alter its interaction with proteins involved in transcriptional co-activator pathways, such as the loss of mediator-associated CDK activity [17, 18]. MED12 has also been shown to interact directly with P300, a PR co-activator and histone acetyltransferase, to maintain an active state for the enhancer [44]. Detailed studies are underway to determine whether and how mut-MED12 alters genome-wide PR binding in LM cells to potentially discover the mechanisms of LM tumorigenesis.

In summary, our findings suggest that DNA methylation, histone modification, and MED12 mutation status together constitute a complex regulatory network that influences P4/PR-mediated RANKL transcription. Using RANKL as a representative PR target gene, our study provides novel and important insights into the regulation of PR action and provides rationale for further elucidation of P4/PR-mediated transcriptional regulation in LM. Dissecting the relationship among TF binding, histone modification, and DNA methylation will improve our understanding of the mechanisms underlying LM development. Considering the heterogeneous genetic background of LM pathogenesis, our studies also represent a key step towards a better understanding of mechanisms underlying the pathogenesis of specific LM subtypes and the necessity for personalized therapeutic strategies.

## Materials and methods

### Tissue collection

Northwestern University’s Institutional Review Board approved the use of human tissue. LM and matched MM tissues were obtained from premenopausal women undergoing either myomectomy or hysterectomy (age 38 ± 9 years, range 28–49 years). Patients receiving hormone treatment 6 months prior to surgery were excluded. Tissues were dissociated and cells isolated as previously described [11].

### Primary cell and tissue explant culture

Primary cells and ex vivo explant culture of MM and LM tissue were performed as previously described [9, 11]. For 5ʹ-aza treatment experiments, if not indicated in the figures, cells were treated with 5 µM 5ʹ-aza for 96 h with medium refreshed every 24 h (A3656; Sigma-Aldrich, St Louis, MO, USA). Tissue explants were treated with vehicle or R5020 in the presence or absence of RU486 (10^−6^ M) for 48 h. To elucidate the role of RANKL in LSC function, the explants were subjected to vehicle or RANKL (CYT-334; ProSpec Bio, Rehovot, Israel) treatment for 24 h and analyzed using flow cytometry.

### Stem cell culture

Each freshly FACS-sorted population of LM cells was cultured in mesenchymal stem cell growth medium (PT-3238; Lonza, Basel, Swizerland) in low-attachment 96-well plates (07-201-680; Fisher Scientific, Chicago, IL, USA) to maintain stem cell characteristics. After 5 days, cells were starved for 24 h using basal mesenchymal stem cell growth medium followed by treatment with RANKL or vehicle for 24 h. Apoptosis assay was performed using Annexin V-FITC Apoptosis Detection Kit (ab14085; Abcam, Cambridge, UK) on FACS-sorted cells treated with vehicle or RANKL for 24 h and analyzed using flow cytometry. Proliferation assay was performed using Cell Counting Kit-8 (CK04-05; Dojindo, Rockville, MD, USA).

### PR siRNA knockdown

LM passage zero cells were transfected with two different PR siRNAs (D-003433-03-0010 and D-003433-01-0010; Dharmacon, Lafayette, CO, USA) or control siRNA (D-001810-10-05) for 96 h, and stimulated with vehicle or R5020 for 24 h before harvesting.

### Antibodies and primers

All antibodies and primers used in this study are listed in Supplementary Table 3.

### Antibody-based cell sorting

CD34^+^/CD49b^+^, CD34^+^/CD49b^−^, and CD34^−^/CD49b^−^ LM cells were FACS-sorted as previously described [19].

### Real-time PCR

About 0.2–1 µg of total RNA was reverse transcribed and quantified using real-time PCR as previously described [11].

### Immunohistochemistry

Paraffin-embedded LM and MM tissues were sectioned and immunohistochemistry was performed by the Northwestern University Pathology Core Facility to detect RANKL expression as previously described [11]. The score was independently calculated by two individuals who were blinded to the treatment group allocation.

### Chromatin immunoprecipitation assay

About 0.2–0.5 g of frozen LM and matched MM tissues were used for ChIP using the SimpleChIP Kit (#9005; Cell Signaling Technology, Danvers, MA, USA). Chromatin was isolated and incubated with antibodies against PR, MED12, H3K4me3, H3K27Ac, or H3K27me3. Normal rabbit IgG was used as a negative control.

### MethylCap-qPCR

Genomic DNA was extracted from LM and MM tissues or cells using DNeasy Blood and Tissue Kit (69504; Qiagen, Hilden, Germany) and fragmented to 300–500 bp using Covaris M220 (Covaris, Woburn, MA, USA). Methylated DNA fragments were captured using the MethylCap Kit (C02020010; Diagenode, Denville, NJ, USA).

### 3C-qPCR

3C assay was performed and analyzed following previously published protocol [45]. Briefly, LM and MM primary cells were fixed with 2% formaldehyde. Nuclei were digested using *PciI* (400 U, 50,000 U/ml) (New England Biolabs, Ipswich, MA, USA) followed by ligation with T4 ligase (M0202L, New England Biolabs) in diluted condition. Ligated DNA was then de-cross-linked (overnight at 65 °C) and purified by classical phenol extraction procedures. All primers were designed within 150 bp of *PciI* sites and detailed information is provided in Supplementary Table 3. A control template containing all ligation products in equal amounts was used to optimize qPCR. All results were normalized using a primer set located in GAPDH gene [50]. To test product purity, we sequenced the 3C-PCR product using Sanger sequencing.

### Next-generation sequencing and analysis

Next-generation sequencing libraries for MethylCap-Seq and PR ChIP-Seq were prepared using the KAPA Hyper Prep Kit (KK8502; KAPA Biosystems, Wilmington, MA, USA) and KAPA Single-Indexed Adapter Kit (KK8710, KAPA Biosystems). The libraries were sequenced at the Northwestern University NUSeq Core Facility using the NextSeq 500 system (Illumina, San Diego, CA, USA) with 20–30 million reads per sample (75 bp single-end for PR ChIP-Seq and 75 bp paired-end for MethylCap-Seq). Sequences were aligned to the hg19 reference genome using Bowtie2. We performed following analysis using Homer: peak calling (-style factor for PR ChIP-Seq and -style histone for MethylCap-Seq), differential binding analysis (getDifferentialPeaks) and motif analysis (findMotifsGenome.pl) [46]. Pathway enrichment analysis was performed using Metacore V6.34 (Thomson Reuters). Sequencing tracks were visualized using UCSC Genome Browser. Visualization of DNA methylation at the PR-binding regions was performed using NGSplot [47]; the methylation level at PR-binding regions was quantified using normalized RPKM value. The GEO accession number for the sequencing data reported in this paper is GSE113108.

### Immunoprecipitation

About 0.2–0.5 g of frozen LM and MM tissues were lysed in non-denaturing buffer (0.5% NP-40, 50 mM Tris-HCl pH 8.0, 250 mM NaCl, 5 mM EDTA) containing protease inhibitor (11836170001; Sigma-Aldrich) for 2 h at 4 °C. One hundred and fifty microgram protein lysate was used for IP following previously published protocol [48]. Five percent of input was used as a loading control. Proteins were boiled for 10 min and separated by SDS-PAGE using precast 4–12% Bis-Tris gels (Thermo Fisher Scientific; NP0315BOX). IP with normal rabbit IgG was used as a negative control.

### Immunoblotting

Whole-cell extracts were prepared and western blot analysis was performed as previously described [11]. ImageJ was used to qualify the intensity of blots.

### Luciferase assay

The RANKL gene distal cis-regulatory element spanning the novel DMR and distal PRBS (RDRE) was amplified by PCR using primers listed in Supplementary Table 3 and cloned into a CpG-free luciferase construct hCpGL-EF1A-Basic [22, 49]. The plasmid was in vitro methylated and tested as described previously [49]. LM and MM primary cells were transfected with 2 μg reporter plasmid and 0.2 μg PRL-TK-Luc using Lipofectamine 3000 Reagent (L3000008; Fisher Scientific). Cell extracts were prepared using passive lysis buffer (E1910; Promega, Madison, WI, USA). Results were normalized to PRL-TK-Luc using a dual luciferase reporter assay system.

### Statistical analysis

All of the statistical analysis in this study were performed using GraphPad Prism 7 (GraphPad Inc., La Jolla, CA, USA) with detailed statistic test description in each figure legend. Sample size was determined based on previous publications [11, 19]. No sample was excluded during analysis. Values were considered statistically significant when *P* < 0.05. Similarity of variance across compared groups was tested by: F-test in two groups, Sum of Square in multiple groups. Normal distribution of data was assessed by Shapiro–Wilk test. Error bar represented SEM for biological replicates of three technical replicates within one patient. All experiments are repeated with at least three patients, with patient number stated in the figure legend.

## Supplementary information

Supplementary Figure Legend

Supplemantary Figure S1

Supplementary Figure S2

Supplementary Figure S3

Supplementary Figure S4

Supplementary Table 1_PR_ChIP-Seq_LM_peaks_bedfile

Supplementary Table 2_PR_ChIP-Seq_LM_higher_peaks_bedfile

Supplemantary Table 3_Antibody_Primer_Info
