# Supplementary material for: Progesterone receptor integrates the effects of mutated MED12 and altered DNA methylation to stimulate RANKL expression and stem cell proliferation in uterine leiomyoma
Source: Oncogene. 2018 Dec 11;38(15):2722–35. doi: 10.1038/s41388-018-0612-6 (PMC6461478; doi:10.1038/s41388-018-0612-6)
Supplement: Supplementary file 1 — Supplementary Figure Legend [file 41388_2018_612_MOESM1_ESM.pdf]

Supplementary Figure S1 Apoptosis level after RANKL treatment in each LM cell population. **A)** Scatter graph showing apoptosis of each LM cell population after 24h incubation with vehicle (0.1% BSA in PBS) or RANKL (100 ng/ml) (means ± SEM, n=3, paired t-test). LM cells were stained with anti-Annexin V and PI (50μg/ml) and analyzed by the LSR Fortessa system. **B)** Representative FACS scattergram for panel A.

Supplementary Figure S2. Differential RANKL regulation by PR signaling in MM and LM.

A) Left panel: Bar graph showing qPCR results of RANKL mRNA levels in LM cells transfected with 2 different PR siRNAs (siPR-1 and siPR-2) or scrambled control siRNA (siCtrl) for 96 h followed by treatment with or without R5020 for 24h. (means ± SEM, n=4, ***P<0.001 and ****P<0.0001, paired two-way ANOVA). Right panel: Representative immunoblot showing PR level with or without PR knockdown. **B**) Bar graph showing the validation results of PR ChIP-Seq. Primers were designed to target 3 DNA fragments around the RANKL distal PR-binding sites: -87,360bp/-87,185bp (chr13:43,060,931-43,061,106), -87,024bp/-86,855bp (chr13:43,061,267-43,061,436) , and -86,884bp/-86,731bp (chr13:43,061,407-43,061,560) upstream of the RANKL transcription start site. (means ± SEM, *P<0.05, paired two-way ANOVA, n=3). **C)** Scatter plot showing PR enrichment at the RANKL distal PRBS positively correlates with PR enrichment at the RANKL promoter (n=9, R^2=0.7407, **P<0.01, Pearson correlation). **D)** Bar graph showing IgG and PR enrichment at the RPL30 gene. (means ± SEM, n=3) **E**) Relative PR mRNA level in MM and LM tissue (means ± SEM, n=13, **P<0.01, paired Student t-test). **F**) Immunoblot showing PR protein levels in chromatin isolated from LM and MM tissues from 4 patients. H3 was used as loading control of chromatin. PT: patient.

Supplementary Figure S3. Effects of DNA methylation on RANKL/RANK pathway. Bar graphs showing mRNA levels of RANK (**A**) and OPG (**B**) genes in primary MM and LM cells treated with or without 5'-aza for four days. (means ± SEM, n=3) **C**) Bar graph showing RANKL mRNA levels treated with 5'-aza (5μM, 4 days) in the presence or absence of cycloheximide (100 μg/ml, 24h). N.S. = not significant. (means ± SEM, n=3) **D**) Bar graph showing MethylCap-qPCR analysis of the DNA methylation levels of RDRE (-88,092bp/-87,961bp, Left panel) and the RANKL promoter (-1,256bp/-1,118bp, Right panel) in MM and LM primary cells treated with or without 5'-aza (5μM) for four days (means ± SEM, n=4, **P*<0.05 and ***P*<0.01, paired two-way ANOVA). **E**) Bar graph showing methylation ratio (Beta value of HumanMethylation450K Array) of the RANKL promoter methylation level in MM and LM primary cells treated with or without 5'-aza (5μM) for four days. (means ± SEM, n=3, paired two-way ANOVA). Each number on the X-axis indicated a CpG probe on HumanMethylation450K Array near RANKL promoter region. **F**) Bar graph showing MethylCap-qPCR result of RDRE in each FACS-sorted LM cell population (means ± SEM, n=4, **P*<0.05 and ***P*<0.01, paired one-way ANOVA). **G**) Bar graph showing qPCR result of RANKL expression levels in each FACS-sorted LM cell population treated with or without 5'-aza for 96h. (means ± SEM, n=3, ***P*<0.01, paired t-test).

Supplementary Figure S4. Influence of MED12 mutation on the compositions of LM cell populations. A) Representative FACS scattergram showing LM cell population distribution. Cells isolated from WT LM and G44D LM tissues were stained with anti-CD45

(depleted hematopoietic cells), anti-CD34, anti-CD49b, and PI (depleted dead cells) and analyzed by the LSRFortessa system. B) Scatter graph showing quantification of the percentage of each LM cell population showed in A.
