# Supplementary figures and images for: Progesterone receptor integrates the effects of mutated MED12 and altered DNA methylation to stimulate RANKL expression and stem cell proliferation in uterine leiomyoma
Source: Oncogene. 2018 Dec 11;38(15):2722–35. doi: 10.1038/s41388-018-0612-6 (PMC6461478; doi:10.1038/s41388-018-0612-6)

# Supplementary Figure S1

A

● Veh  
■ RANKL

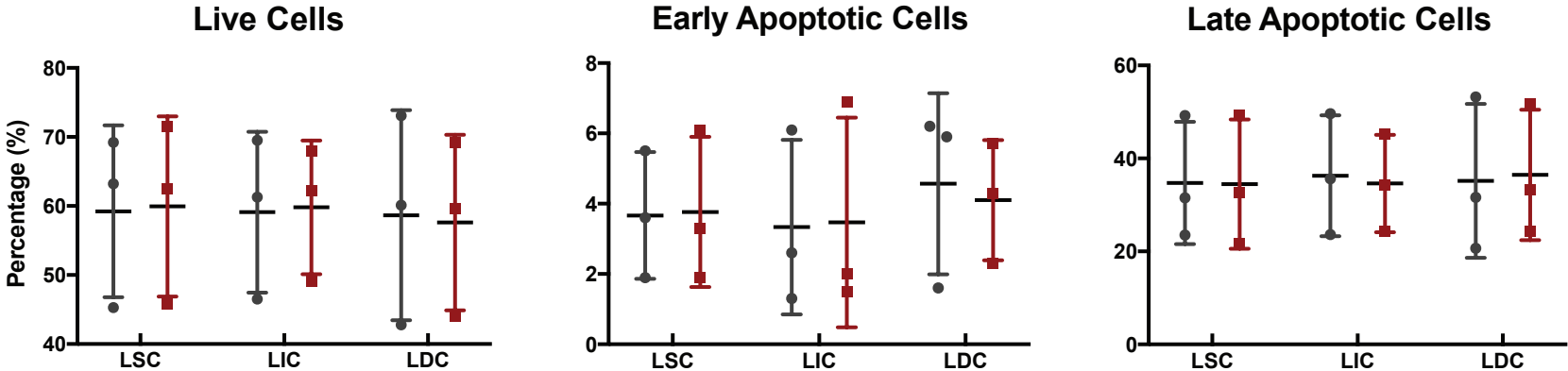

B

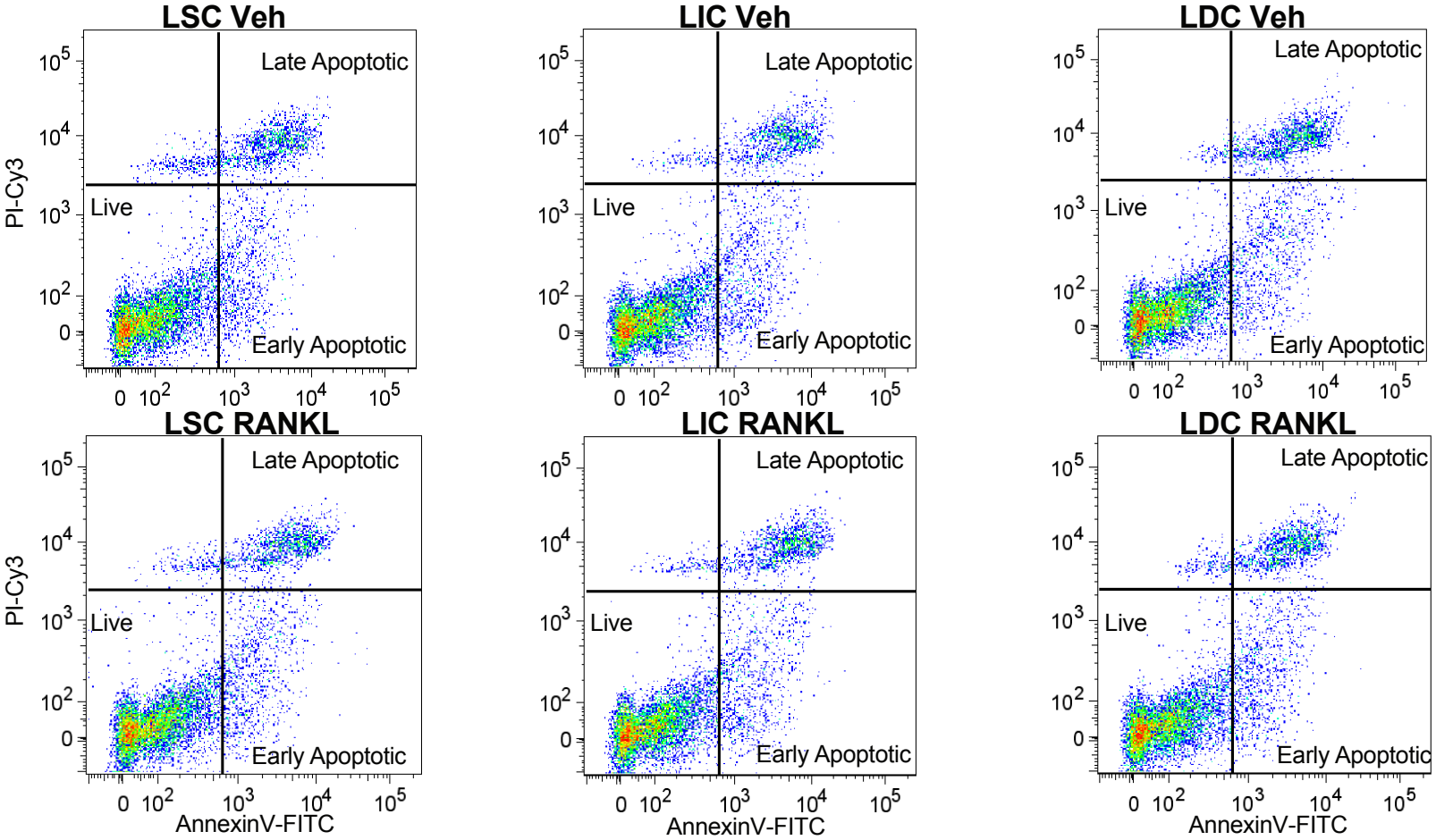

Supplement: Supplementary file 2 — Supplemantary Figure S1 [file 41388_2018_612_MOESM2_ESM.pdf]

# Supplementary Figure S2

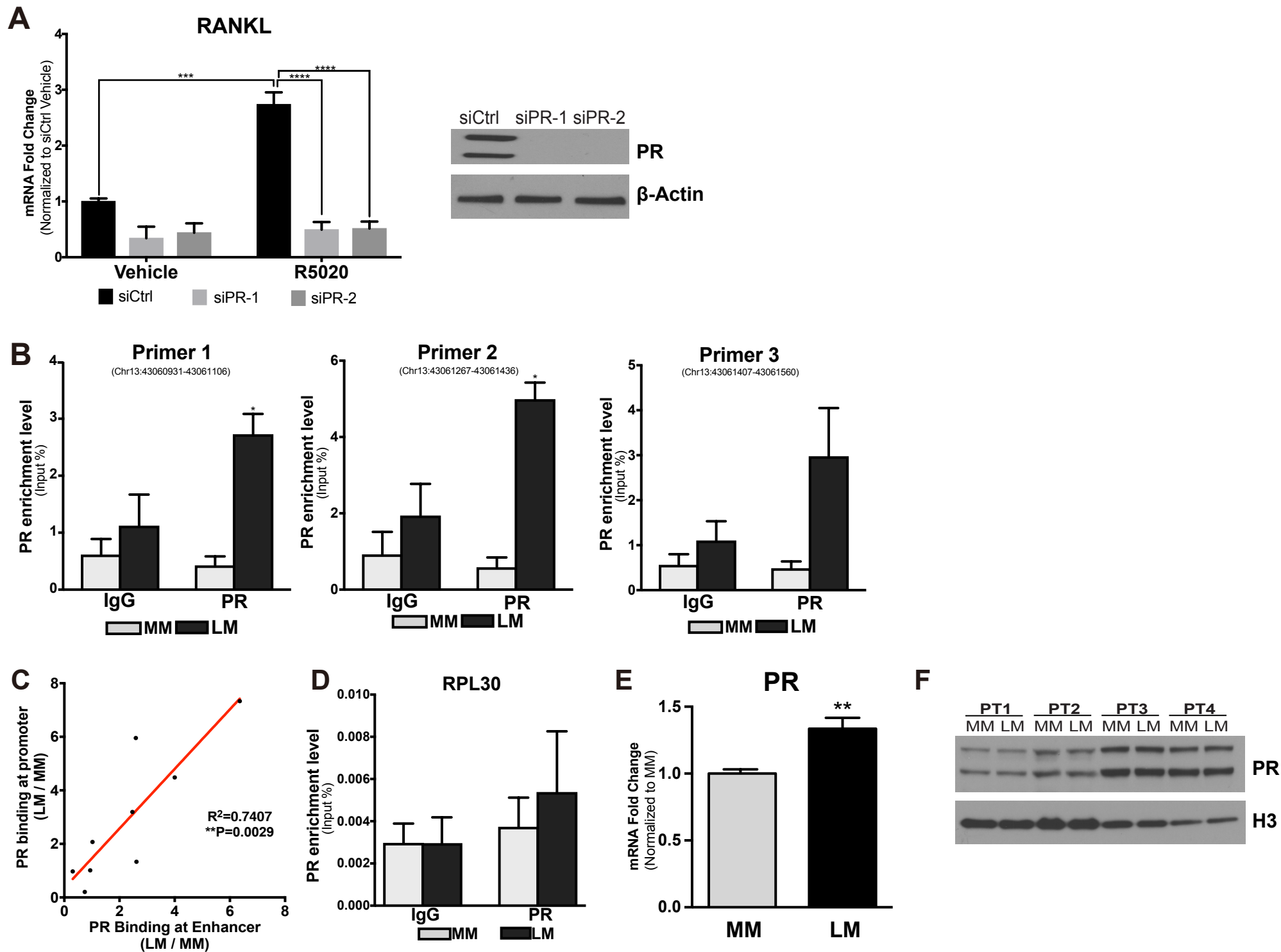

Supplement: Supplementary file 3 — Supplementary Figure S2 [file 41388_2018_612_MOESM3_ESM.pdf]

# Supplementary Figure S3

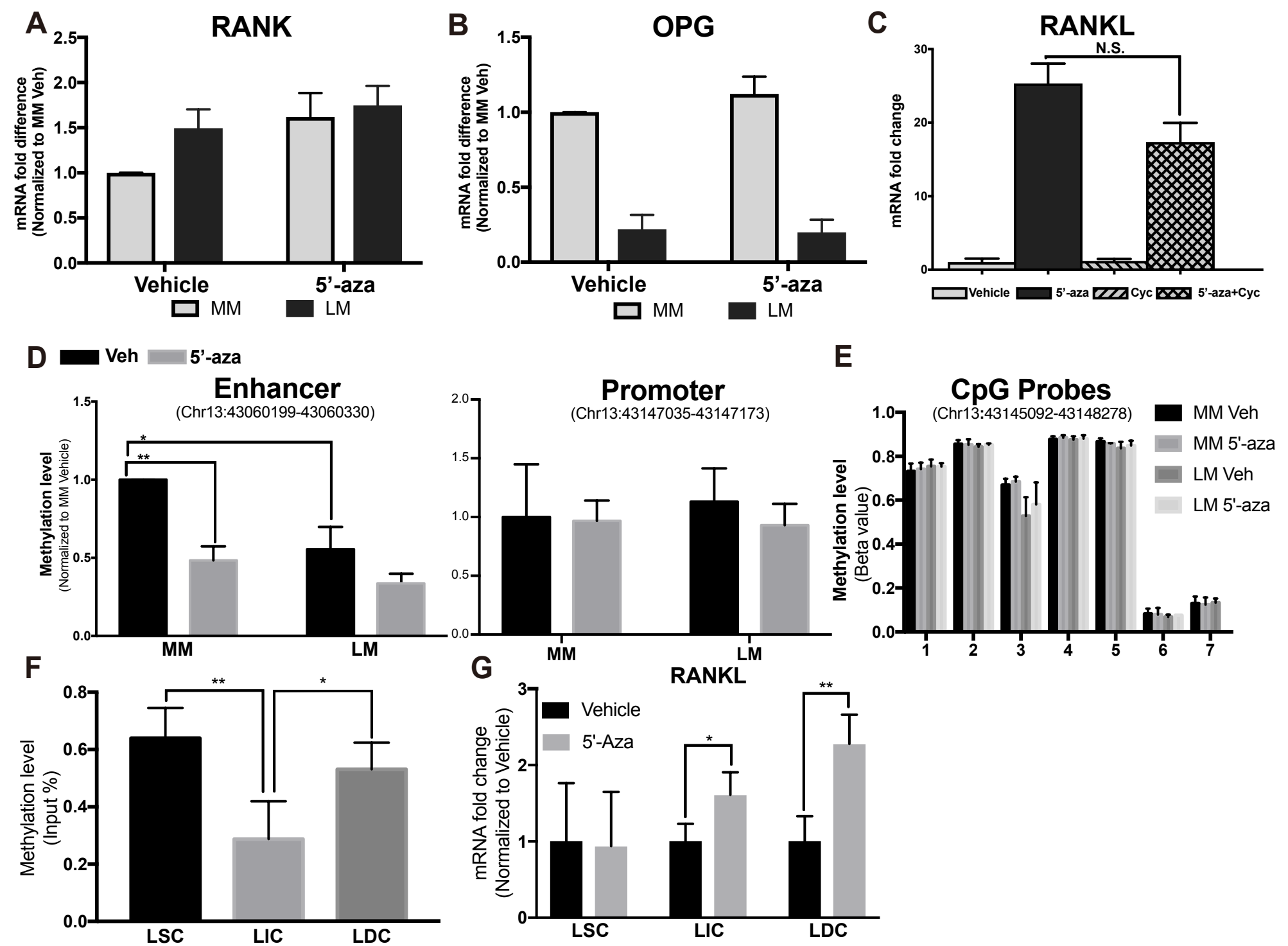

Supplement: Supplementary file 4 — Supplementary Figure S3 [file 41388_2018_612_MOESM4_ESM.pdf]

Supplementary Figure S4

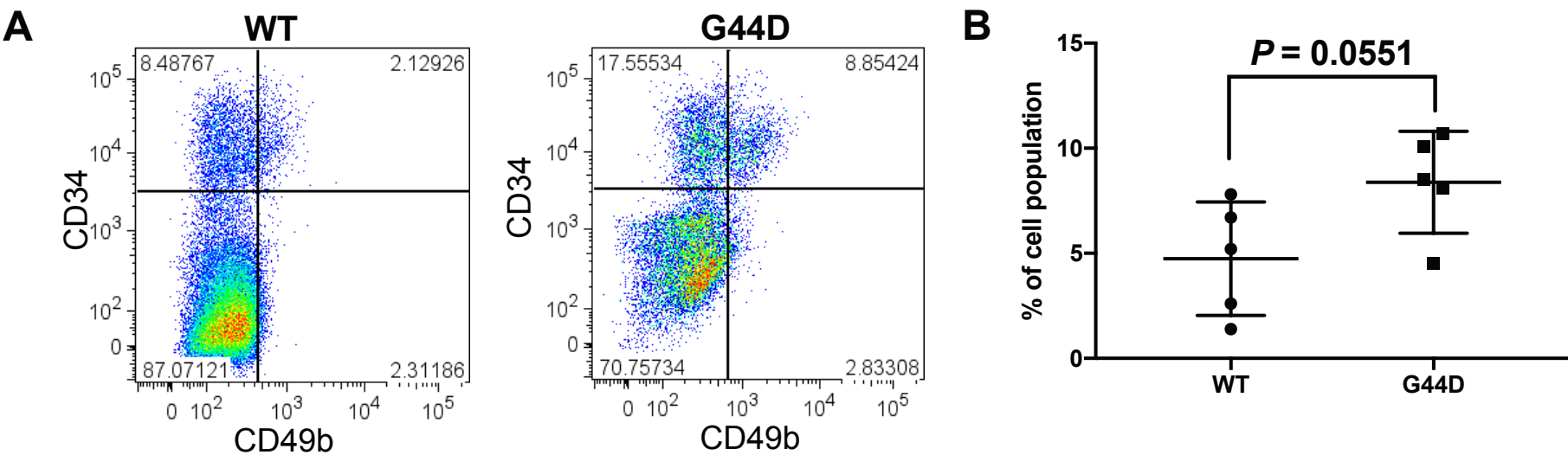

Supplement: Supplementary file 5 — Supplementary Figure S4 [file 41388_2018_612_MOESM5_ESM.pdf]
